# Sleep and allergic diseases among young Chinese adults from the Singapore/Malaysia Cross-Sectional Genetic Epidemiology Study (SMCGES) cohort

**DOI:** 10.1186/s40101-024-00356-5

**Published:** 2024-01-30

**Authors:** Qi Yi Ambrose Wong, Jun Jie Lim, Jun Yan Ng, Yi Ying Eliza Lim, Yang Yie Sio, Fook Tim Chew

**Affiliations:** Department of Biological Sciences, Faculty of Science, National University of Singapore, Allergy and Molecular Immunology Laboratory, Lee Hiok Kwee Functional Genomics Laboratories, Block S2, Level 5, 14 Science Drive 4, Lower Kent Ridge Road, Singapore, 117543 Singapore

**Keywords:** Allergic rhinitis, Atopic dermatitis, Asthma, ISAAC, Sleep

## Abstract

**Background and objective:**

Sleep disruption has been shown to affect immune function and thus influence allergic disease manifestation. The specific effects of sleep on allergic diseases, however, are less well-established; hence, in a unique population of young Chinese adults, we investigated the association between sleep and allergic disease.

**Methods:**

Young Chinese adults recruited from Singapore in the Singapore/Malaysia Cross-Sectional Genetic Epidemiology Study (SMCGES) were analyzed. We used the International Study of Asthma and Allergies in Childhood (ISAAC) protocol and a skin prick test to determine atopic dermatitis (AD), allergic rhinitis (AR), and asthma status. Information regarding total sleep time (TST) and sleep quality (SQ) was also obtained.

**Results:**

Of 1558 participants with a mean age of 25.0 years (SD = 7.6), 61.4% were female, and the mean total sleep time (TST) was 6.8 h (SD = 1.1). The proportions of AD, AR, and asthma were 24.5% (393/1542), 36.4% (987/1551), and 14.7% (227/1547), respectively. 59.8% (235/393) of AD cases suffered from AD-related sleep disturbances, 37.1% (209/564) of AR cases suffered from AR-related sleep disturbances, and 25.1% (57/227) of asthma cases suffered from asthma-related sleep disturbances. Only asthma cases showed a significantly lower mean TST than those without asthma (*p* = 0.015). Longer TST was significantly associated with lower odds of AR (OR = 0.905, 95% CI = 0.820–0.999) and asthma (OR = 0.852, 95% CI = 0.746–0.972). Linear regression analyses showed that lower TST was significantly associated with asthma (*β* =  − 0.18, SE = 0.076, *p*-value = 0.017), and AR when adjusted for AR-related sleep disturbances (*β* =  − 0.157, SE = 0.065, *p*-value = 0.016). Only sleep disturbances due to AR were significantly associated with a poorer SQ (OR = 1.962, 95% CI = 1.245–3.089).

**Conclusions:**

We found that sleep quality, but not sleep duration was significantly poorer among AD cases, although the exact direction of influence could not be determined. In consideration of the literature coupled with our findings, we posit that TST influences allergic rhinitis rather than vice versa. Finally, the association between TST and asthma is likely mediated by asthma-related sleep disturbances, since mean TST was significantly lower among those with nighttime asthma symptoms. Future studies could consider using objective sleep measurements coupled with differential expression analysis to investigate the pathophysiology of sleep and allergic diseases.

**Supplementary Information:**

The online version contains supplementary material available at 10.1186/s40101-024-00356-5.

## Introduction

### Background

The circadian clock in humans is characterized by an approximately 24-h period which persists in the absence of external time cues and manifests as oscillatory patterns in physiological parameters such as body temperature, hormone levels, and metabolism [1–3]. These rhythms are synchronized by the master circadian clock—two suprachiasmatic nuclei (SCN) located in the anterior hypothalamus [3]. In turn, the master clock is entrained to environmental cues by zeitgebers such as light, food intake, temperature, and physical activity [4, 5]. Light, the most potent zeitgeber, acts via the intrinsically photosensitive retinal ganglion cells (ipRGC) in the eyes to entrain the SCN to the light–dark cycle [2, 6–8]. Thus, in a circadian rhythm synchronized to the light–dark cycle, the SCN regulates the synthesis of melatonin which not only facilitates the regulation of the circadian rhythm, but has also been linked to various physiological functions, including immune response [9–12]. Indeed, evidence has been reported for the regulation of IL-2 and IL-6 by melatonin via the retinoid-related orphan nuclear hormone receptor family (RZR/ROR) [13, 14]. Moreover, the discovery of RZR and ROR in other immune cell types, i.e., monocytes, B-cells, T-cells, and NK cells, has further raised the possibility of a wider influence of melatonin and thus the circadian rhythm on immune response [15]. Phenotypically, the circadian rhythm has been implicated in the 24-h cycle in AR symptom manifestation and severity, wherein the mechanism has yet to be determined [16]. Additionally, nocturnal bronchial asthma has been attributed to the release of pro-inflammatory cells in a response to daytime antigen exposure, resulting in asthma exacerbation at the end of the day [16].

The most salient effect of the circadian rhythm on human behavior is the sleep–wake cycle [17]. Sleep and wake states are generated by neural networks governed by circadian rhythms entrained to the light–dark cycle, resulting in a sleep–wake cycle coinciding with the 24-h day [18]. Importantly, sleep has been established to be a biological imperative essential to physiological and psychological well-being, the disruption of which not only affects circadian rhythmicity, but also predisposes the individual to an increased risk of disease, such as cardiovascular disease, diabetes, and cancer [19–22]. As is the case with the circadian rhythm, sleep has also been linked to immune function [23, 24]. The impairment of either sleep duration or sleep quality is associated with a reduced antibody production and increased generation of inflammatory cytokines [18, 25]. Hence, the dual association of the circadian rhythm and sleep with immune function has implications for the pathophysiology of allergic disease such as atopic dermatitis (AD), allergic rhinitis (AR), and asthma [26–28]. Notably, the disentanglement of the effects of the circadian rhythm and sleep on immune function is challenging given the tight intertwinement of the circadian rhythm and sleep [23].

Notwithstanding, a correlation between sleep and allergic diseases has been observed in several epidemiologic studies. Results from the National Health and Nutrition Examination Survey (NHANES) showed that impaired sleep resulting from obstructive sleep apnea was associated with higher odds of hay fever and eczema, while a sleep duration of 6 or fewer hours correlated with higher odds of allergic sensitization [29]. Studies focusing specifically on sleep and AD-related outcomes consistently found AD to be significantly associated with sleep quality, but not with sleep duration [30–32]. Interestingly, although increased AD severity was also significantly associated with more sleep disruption, there was an increased likelihood of sleep disturbance among those with mild or inactive AD [31]. Moreover, sleep disruptions among individuals with AD in remission were not accompanied by nighttime itching episodes [33]. Investigations of AR and sleep showed that increased upper airway resistance and nasal discharge, both of which are symptoms characteristic of AR, caused microarousals during sleep [34]. Furthermore, increased upper airway resistance associated with AR was accompanied by obstructive sleep apnea and sleep-disordered breathing, which also negatively impacted sleep quality [35, 36]. Conversely, a recent systematic review found that sleep duration was not significantly associated with AR, while sleep quality score, sleep disturbance scores, and sleep latency scores were higher among AR patients; sleep efficiency was nonetheless decreased among AR patients [37]. Finally, poor sleep patterns have been found to increase asthma risk [38]. While asthma has been associated with poor sleep quality, this was likely the result of asthma symptoms affecting sleep, or comorbid AR resulting in AR-related sleep disturbances in asthmatics [39, 40].

Despite the putative evidence for sleep influencing allergic disease risk, the consensus that sleep can be disturbed by allergic diseases and that sleep disturbances can be used as an indicator of allergic disease severity introduces a dimension of confoundment to the directionality of the relationship between sleep and allergic disease [24]. Indeed, an increased prevalence and severity of sleep disturbances were found to be associated with increased AD severity as determined by SCORing Atopic Dermatitis (SCORAD) [41]. In AR, Allergic Rhinitis and its Impact on Asthma (ARIA) defines moderate-severe AR as AR which negatively impacts any aspect of quality of life, including sleep disturbances [42]. Furthermore, systematically reviewed evidence indicates that AR is associated with decreased sleep duration and quality, sleep-related disorders, and a resultant daytime dysfunction due to sleep impairment [37]. Lastly, nighttime asthma awakening is essential for the assessment of asthma severity, control, and remission [43–46].

### Objectives

Presently, the preponderance of Asian studies assessing sleep and allergic disease originates from the Korea National Health and Nutrition Examination Survey (KNHANES) [47–49]. As such, there is a paucity of epidemiologic studies assessing the relationship between sleep and allergic disease among the wider Asian population. Moreover, many epidemiologic studies thus far have been remiss to address a chicken-and-egg dilemma: what is the direction of the relationship between sleep and allergic disease, i.e., does sleep influence allergic disease manifestation, or vice versa?

Here, we have selected young Chinese adults recruited in Singapore from the Singapore/Malaysia Cross-Sectional Genetic Epidemiology Study (SMCGES) database with the consideration that majority of the Singapore subjects were Chinese (86.8%). Moreover, we note that the Chinese population constitutes a major distinct ethnic group both in Asia and worldwide [50, 51]. Despite the predominance of Chinese, sleep and allergic disease have not been studied in this ethnic group in Asia. Of an additional consideration, our focus on Chinese subjects also aims to reduce ascertainment bias resulting from the small proportions of non-Chinese ethnicities and counteract potential confounding introduced by the inclusion of non-Chinese ethnicities who feature differing genetic characteristics. Using the International Study of Asthma and Allergies in Childhood (ISAAC) questionnaire, which comprises utilities for collecting data on sleep duration and sleep disturbances resulting from allergic disease, we have performed our data collection and case classification according to internationally established standards [52].

In summary, our present analysis focuses on a novel group of young Chinese adults recruited in Singapore with three aims: (i) establish a baseline duration of sleep among our subjects, (ii) perform an exploratory investigation of the association between sleep and allergic disease, and (iii) assign directionality to the relationship between sleep and allergic disease by accounting for sleep disturbances due to allergic disease.

## Methods

### Participants and data collection

The Singapore/Malaysia Cross-Sectional Genetic Epidemiology Study (SMCGES) is an ongoing large-scale cross-sectional study. Utilizing email and poster advertisements, participants were recruited across the campuses of the National University of Singapore (NUS), Singapore; Universiti Tunku Abdul Rahman, Malaysia; and Sunway University, Malaysia. Participants aged at least 18 years old and consenting to participate in the study completed an investigator-administered skin prick test (SPT) and an adapted ISAAC survey.

The SPT was performed after verifying that the participant had not consumed antihistamines for at least 3 days preceding the test; participants who had done otherwise were rescheduled. Participants were assessed for sensitization to *Blomia tropicalis* and *Dermatophagoides pteronyssinus*: two dust mite species selected due to their high prevalence in Singaporean homes and high rates of sensitization among the local atopic population [53, 54]. A positive SPT result, indicating sensitization, was defined as the development of a wheal of at least 3 mm in diameter in response to any of the two allergens. In addition, a positive histamine control and negative saline control were included, consistent with the standard SPT protocol used for the SMCGES thus far [55]. Subjects showing a positive SPT result were classified as atopic cases.

Our survey was adapted from the published ISAAC Phase Three questionnaire which has been standardized and validated for the assessment of allergic diseases internationally [56]. The survey was administered according to established ISAAC protocol and yielded data on allergic disease symptoms, sleep disturbances due to allergic disease, and epidemiology. Additionally, subjects were queried for an estimate of their total sleep time (TST) and their perception of their sleep quality (SQ).

The present report concerns Chinese subjects recruited during data collection exercises in NUS, Singapore, which has occurred in August annually since 2005. Subjects excluded from the current analysis fulfilled any of the following criteria: (i) recruited in Malaysia, (ii) non-Chinese ethnicity, or (iii) did not provide their estimated total sleep time.

### Allergic disease classification

Using data obtained via the ISAAC questionnaire, subjects’ disease statuses were determined for AD, AR, and asthma. Among atopic subjects identified by the SPT, AD cases comprised those who had suffered from a recurrent itchy rash for at least 6 months in any of the specified anatomical locations: flexural region of the elbows and knees, front of the ankles, under the buttocks, around the neck, cheeks, eyes, or ears. Per ARIA 2008 guidelines, atopic subjects reporting at least two rhinitis symptoms out of nasal blockage, nasal pruritus, sneezing, and rhinorrhea when they were not afflicted with a cold or flu were identified as AR subjects [42]. Finally, subjects who had ever had asthma were classified as asthma cases. The classification criteria were consistent with that of previous reports [55, 57, 58].

### Sleep disturbances due to allergic disease

For AD, AR, and asthma, the respective sleep disturbances were investigated. Among AD cases, phenotypes considered to entail sleep disturbances (*AD-related sleep disturbances*) included having ever been kept awake by an itchy rash, itching in the evening or night, and itching that impacted sleep in general. In AR, subjects reporting any of the four rhinitis symptoms (see the section above) to be severe enough to interfere with sleep or indicating general sleep disturbance due to AR symptoms were considered to have sleep disturbances due to AR (*AR-related sleep disturbances*). Finally, asthma-afflicted subjects who had either suffered from sleep disturbances due to wheezing, had a dry nocturnal cough not related to a cold or chest infection, or experienced nighttime asthma attacks were classified as individuals having *asthma-related sleep disturbances*.

### Statistical analyses

Data was compiled using Microsoft Excel. Data cleaning and statistical analyses were performed using R software, version 4.0.3 [59]. Welch’s *t*-tests were conducted for continuous variables while Pearson’s chi-squared tests were conducted for categorical variables. Finally, linear regression models were constructed to assess the impact of allergic diseases and sleep disturbances due to allergic diseases on total sleep time; unadjusted and adjusted analyses were performed, with the latter accounting for sleep disturbance due to allergic disease. Logistic regression was performed to evaluate binary outcomes, namely, allergic disease status against total sleep time and sleep quality, and sleep quality (very bad to moderate vs good to very good) against allergic disease status and sleep disturbances. Statistical significance was determined where the accompanying *p*-value was below 0.05 (*p* < 0.05).

## Results

### Sample description

**Fig. 1:**
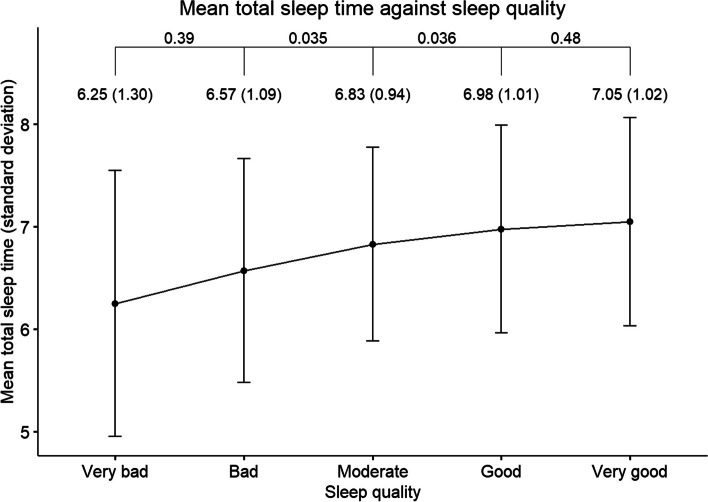
Mean total sleep time and accompanying standard deviation, in hours, for each grade of sleep quality

Of 13,410 subjects ascertained in the Singapore cohort of the SMCGES, a subset was administered the sleep questions; our current analysis focuses on 1558 young Chinese adults who provided an estimate of their sleep duration. The mean age of our subjects was 25.0 years (SD = 7.6 years) and 61.4% were females. Overall, the mean total sleep time (TST) of our sample was 6.8 h (SD = 1.1 h). Most respondents reported having a good quality of sleep in general (43.2%). There was a significant decrease in mean TST from good, to moderate, to bad SQ (Fig. 1). Conversely, there was no significant difference between the TST of good versus very good sleep, nor bad versus very bad sleep. The proportions of AD, AR, and asthma among the sample subjects were 25.5% (393/1542), 36.4% (987/1551), and 14.7% (227/1547), respectively. A breakdown of sample demographics and sleep characteristics by allergic disease status is summarized in Table 1.

**Table 1:** Summary of demographic and sleep characteristics for the overall sample and by allergic disease status

**Variable**	**Overall** *N* = 1558^*1*^	**Atopic dermatitis**			**Allergic rhinitis**			**Ever asthma**		
		**No** *N* = 1149^*1*^	**Yes** *N* = 393^*1*^	***p*-value**^*2*^	**No** *N* = 987^*1*^	**Yes** *N* = 564^*1*^	***p*-value**^*2*^	**No** *N* = 1320^*1*^	**Yes** *N* = 227^*1*^	***p*-value**^*2*^
**Age at collection (in years)**	25.0 ± 7.6	25.0 ± 7.9	24.9 ± 6.6	0.769	25.5 ± 8.4	24.0 ± 5.7	< 0.001	24.9 ± 7.6	25.1 ± 7.5	0.697
**Gender**				0.023			0.004			0.006
Female	955 (61.4%)	726 (63.2%)	221 (56.5%)		632 (64.1%)	318 (56.5%)		827 (62.7%)	120 (52.9%)	
Male	601 (38.6%)	423 (36.8%)	170 (43.5%)		354 (35.9%)	245 (43.5%)		491 (37.3%)	107 (47.1%)	
Unknown	2	0	2		1	1		2	0	
**Housing type**				0.010			0.009			0.333
Flats	980 (65.4%)	709 (64.4%)	259 (67.8%)		606 (64.3%)	369 (67.2%)		823 (65.1%)	152 (68.5%)	
Condo/private apartment	408 (27.2%)	321 (29.2%)	86 (22.5%)		278 (29.5%)	129 (23.5%)		346 (27.4%)	59 (26.6%)	
Landed property	110 (7.3%)	71 (6.4%)	37 (9.7%)		59 (6.3%)	51 (9.3%)		96 (7.6%)	11 (5.0%)	
Unknown	60	48	11		44	15		55	5	
**Income category**				0.010			0.005			0.051
< SGD 2000	159 (10.4%)	129 (11.4%)	29 (7.5%)		118 (12.2%)	41 (7.4%)		136 (10.5%)	21 (9.5%)	
SGD 2000—3999	418 (27.4%)	315 (28.0%)	96 (24.9%)		276 (28.6%)	141 (25.4%)		365 (28.1%)	49 (22.3%)	
SGD 4000—5999	338 (22.1%)	256 (22.7%)	80 (20.8%)		206 (21.3%)	131 (23.6%)		292 (22.5%)	43 (19.5%)	
≥ SGD 6000	612 (40.1%)	427 (37.9%)	180 (46.8%)		366 (37.9%)	242 (43.6%)		504 (38.9%)	107 (48.6%)	
Unknown	31	22	8		21	9		23	7	
**Total sleep time (in hours)**	6.8 ± 1.1	6.8 ± 1.0	6.8 ± 1.1	0.480	6.9 ± 1.0	6.7 ± 1.1	0.053	6.8 ± 1.1	6.7 ± 1.0	0.015
**Sleep quality**				0.170			0.933			0.324
Very bad to moderate	453 (44.6%)	328 (43.2%)	118 (48.2%)		303 (44.7%)	147 (44.4%)		390 (44.1%)	61 (48.8%)	
Good to very good	562 (55.4%)	432 (56.8%)	127 (51.8%)		375 (55.3%)	184 (55.6%)		494 (55.9%)	64 (51.2%)	
Unknown	543	389	148		309	233		436	102	

^1^Mean ± SD; *n* (%). ^2^Welch’s two-sample *t*-test was conducted for age and total sleep time. Pearson’s chi-squared test was conducted for housing type and income category

### Associations between sleep and allergic disease

Within the disease case subsets for each allergic disease, 59.8% (235/393) of AD cases suffered from AD-related sleep disturbances, 37.1% (209/564) of AR cases suffered from AR-related sleep disturbances, and 25.1% (57/227) of asthma cases suffered from asthma-related sleep disturbances. The mean TST of individuals afflicted with asthma (6.7 h, SD = 1.1 h) was significantly lower than that of those without (6.8 h, SD = 1.0 h; *p* = 0.015; Table 1). Conversely, neither the mean TST of AD cases versus controls nor AR cases versus controls were significantly different (Table 1). Among those with AD and AR-related sleep disturbances, the mean TST was not significantly different from that of those without sleep disturbances (Table 2). While the mean TST of those with asthma-related sleep disturbance was lower than that of those without, the difference in mean TST was non-significant. Accordingly, the differences in mean TST for those experiencing AD and AR phenotypes resulting in sleep disturbances were non-significant; mean TST was lower among those with asthma-related sleep disturbances than those without, but this difference was likewise non-significant (Table 3).

**Table 2:** Mean total sleep time and accompanying standard deviation, in hours, by presence or absence of sleep disturbances due to allergic disease

**Allergic disease**	**Mean total sleep time (TST) among those with the respective allergic disease**		***p*-value**^*2*^
	No disease-related sleep disturbances^*1*^	Disease-related sleep disturbances^*1*^	
Atopic dermatitis	6.79 ± 1.00 (158)	6.77 ± 1.12 (235)	0.901
Allergic rhinitis	6.70 ± 1.08 (355)	6.82 ± 1.16 (209)	0.198
Asthma	6.71 ± 0.95 (170)	6.50 ± 1.19 (57)	0.226

^1^Mean TST ± SD, in hours (*N*). ^2^Welch’s two-sample *t*-test

**Table 3:** Mean total sleep time and accompanying standard deviation, in hours, by presence or absence of each allergic disease phenotype resulting in sleep disturbance

**Allergic disease phenotype**	**Mean TST among those with or without allergic disease-related sleep disturbance phenotype**		*P***-value**^*2*^
	No ^*1*^	Yes ^*1*^	
**Atopic dermatitis**			
Kept awake by itchy rash	6.79 ± 1.05 (239)	6.77 ± 1.11 (154)	0.841
Evening itch	6.76 ± 1.06 (283)	6.83 ± 1.10 (110)	0.592
Nighttime itch	6.80 ± 0.99 (260)	6.74 ± 1.21 (133)	0.660
Constant itching in the day and night	6.80 ± 1.07 (353)	6.58 ± 1.05 (40)	0.199
Constant itching at night	6.75 ± 1.04 (353)	7.03 ± 1.33 (40)	0.217
Sleep disturbance due to itching	6.78 ± 1.03 (297)	6.78 ± 1.19 (96)	0.970
**Allergic rhinitis**			
Sleep interference due to nasal blockage	6.74 ± 1.11 (420)	6.77 ± 1.08 (28)	0.893
Sleep interference due to nasal pruritus	6.67 ± 1.05 (412)	6.83 ± 1.53 (12)	0.718
Sleep interference due to rhinorrhoea	6.73 ± 1.14 (450)	6.62 ± 0.97 (37)	0.508
Sleep interference due to sneezing	6.74 ± 1.11 (467)	6.67 ± 0.98 (29)	0.723
Sleep disturbances due to AR symptoms	6.68 ± 1.06 (382)	6.87 ± 1.20 (182)	0.065
**Asthma**			
Sleep disturbance due to wheezing	6.98 ± 1.23 (47)	6.57 ± 1.21 (14)	0.282
Dry nocturnal cough	6.71 ± 0.95 (185)	6.45 ± 1.26 (41)	0.219
Nighttime asthma attacks	6.80 ± 1.07 (59)	6.58 ± 1.02 (20)	0.411

^1^Mean ± SD (*N*). ^2^Welch’s two-sample *t*-test

**Fig. 2:**
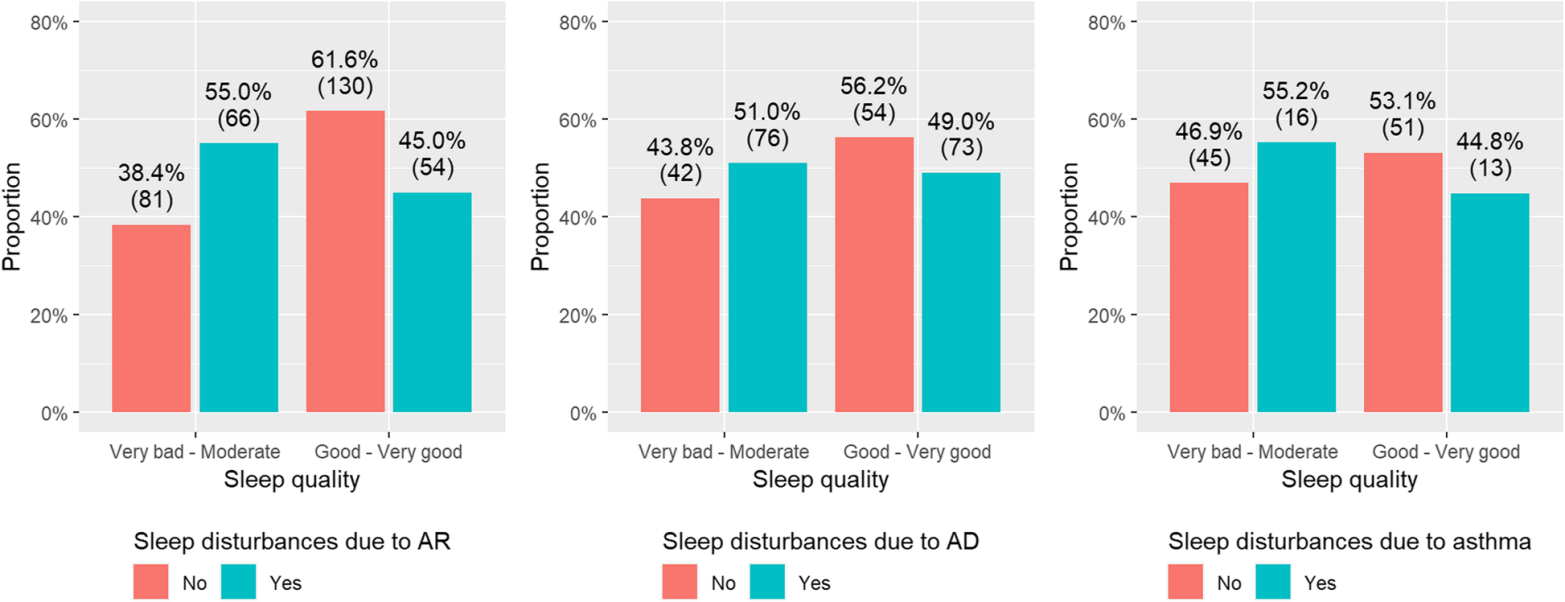
Proportions of sleep quality among allergic disease cases denominated by the presence or absence of sleep disturbances due to each allergic disease

Among AD and asthma cases, there was a lower proportion of respondents experiencing at least a good SQ in general as compared to non-afflicted individuals (i.e., 56.8% of non-AD controls versus 51.8% of AD cases and 55.9% of non-asthma controls versus 51.2% of asthma cases reported having at least a good SQ in general). Where allergic disease cases were concerned, a moderate or worse SQ was reported by 51.0% (76/149) of AD cases suffering from AD-related sleep disturbances, 55% (66/120) of AR cases suffering from AR-related sleep disturbances, and 55.2% (16/29) of asthma cases suffering from asthma-related sleep disturbances (Fig. 2).

### Regression analyses for sleep and allergic diseases

**Fig. 3:**
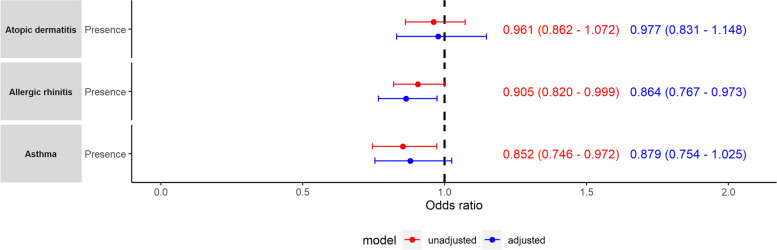
Odds ratios for the presence of AR, AD, and asthma. Adjusted odds ratios were obtained by controlling for the presence of sleep disturbance due to allergic disease

Logistic regression analyses for allergic disease against TST and SQ showed that longer TST was significantly associated with lower odds of AR (odds ratio (OR) = 0.905, 95% CI = 0.820–0.999, *p*-value = 0.048) and asthma (OR = 0.852, 95% CI = 0.746–0.972, *p*-value = 0.017; Fig. 3). Controlled for sleep disturbance due to allergic disease, longer TST was significantly associated with allergic rhinitis only (OR = 0.864, 95% CI = 0.767–0.973, *p*-value = 0.016). SQ was not significantly associated with allergic disease manifestation. The inclusion of both TST and SQ as confounding factors or interaction terms yielded no significant findings. Linear regression modeling of TST against allergic disease and sleep disturbances due to allergic disease showed that AR manifestation was significantly associated with a lower TST, regardless of whether sleep disturbances were included in the model (unadjusted: *β* =  − 0.11, SE = 0.055, *p*-value = 0.047; adjusted: *β* =  − 0.157, SE = 0.065, *p*-value = 0.016), while asthma was significantly associated with lower TST only when unadjusted for sleep disturbances due to asthma (unadjusted: *β* =  − 0.18, SE = 0.076, *p*-value = 0.017). Finally, logistic regression analyses for SQ against allergic disease and allergic disease-related sleep disturbances showed that only sleep disturbances due to AR were significantly associated with a poorer SQ (OR = 1.962, 95% CI = 1.245–3.089, *p*-value = 0.004). The full results from the regression analyses are summarized in Additional file 1: Supplementary Tables 1-4.

## Discussion

### Sleep quality, but not sleep duration, was poorer among AD cases

While a slight majority of AD cases experienced a poorer SQ, we found no significant difference between the mean TST of AD cases versus that of non-AD controls. To an extent, our findings for the lack of difference in sleep duration between AD cases and non-AD controls echo that of previous studies. Indeed, most subjective and objective measurements of sleep duration among pediatric AD cases and controls showed no statistically significant difference [30–32, 60–62]. Among adults, however, reports for sleep duration and AD were few and inconsistent: sleep duration was significantly shorter among AD cases in one NHANES study but non-significantly different elsewhere [63, 64]. Separately, our results correspond to findings that poor SQ was associated with AD. Among both children and adults, AD cases reported various SQ-related impairments, including longer sleep-onset latency, poor sleep efficiency, and longer wakefulness after sleep onset (WASO) [41]. Finally, almost 60% of AD cases reported AD-related sleep disturbances, of which none of the phenotypes were associated with a significant difference in mean TST. These proportions were not unusual for subjective assessments of AD-related sleep disturbances [65, 66]. Additionally, our results provide no evidence that AD is significantly with TST, even in the presence of AD-related sleep disturbances.

Hypotheses for the association of disrupted sleep with AD have implicated pruritus [41, 67]. Sleep disruptions among AD patients have been attributed to scratching motions in response to pruritus [67]. Indeed, Il-31 which has been established as an important mediator of pruritus has also been found to be associated with poorer sleep quality [68, 69]. However, although movements in sleep and worse perception of pruritus was associated with lower sleep quality, arousals resulting from limb movements and scratching were not significantly associated with sleep efficiency [33, 69]. Thus, while pruritus and scratching might be a contributory factor to poor sleep quality among AD cases, they do not constitute the primary etiology [69]. Instead, the circadian rhythm and its effect on immune cell expression have been proposed as a possibility: elevated cytokine and immune cell involved in AD pathogenesis could be involved in regulating sleep [67]. Thus, increased cytokine and immune cell activity at night could be causing AD-related itching, or the dysregulation of cytokines due to AD could be disrupting the circadian rhythm and resulting in impaired sleep [41, 67]. Notwithstanding, the mechanism linking circadian rhythms, immune function and AD has yet to be comprehensively studied and elucidated.

### A shorter sleep duration likely increases the risk of AR

The mean TST among AR cases was significantly lower than that of non-AR controls. At least one-third of AR cases suffered from AR-related sleep disturbances, but the mean TST among sleep-disturbed individuals was not significantly different from the non-sleep-disturbed individuals. Moreover, a small majority of those with AR-related sleep disturbances reported having a poorer SQ with poorer SQ being significantly associated with AR. Concordantly, previous studies have identified a negative impact of AR on SQ assessed using different rubrics [70, 71]. Notably, we identified an association between AR and TST, which remained significant after controlling for AR-related sleep disturbances. Taken together with the current findings that mean TST was not significantly different between sleep-disturbed and non-sleep-disturbed AR cases, our results suggest that TST potentially influences the manifestation of AR as opposed to vice versa. Accordingly, the Korea National Health and Nutrition Examination Survey (KNHANES) study highlighted a similar trend wherein increasing sleep duration was associated with a decrease in the prevalence of AR [49, 72]. Interestingly, the present findings coupled with KNHANES results contrast a recent meta-analysis which found no significant differences in sleep duration between the AR and control groups among reports published before 2019 [37].

There is a dearth of literature examining the mechanisms linking sleep duration and allergic rhinitis. Nonetheless, from studies on circadian rhythm and immune function, disruption of the circadian rhythm precipitated by a lack of sleep has been proposed to influence allergic rhinitis reactions [73]. Additionally, there is evidence that the circadian rhythm regulates AR symptoms, wherein symptoms frequently worsened during the night and in the morning, but subsided in the middle of the day [16]. Immunologically, eosinophil and basophil activities in the nasal mucosa were found to be elevated in the early morning as compared to the afternoon, while the immunoglobulin E (IgE)/mast cell axis has been shown to be controlled by the circadian clock [74–77]. Despite the possible mechanistic links, evidence for a clear pathway between sleep and AR has yet to be clearly illustrated.

### The most apparent consequence of asthma-related sleep disturbances is the impairment of sleep duration

Asthma cases reported a significantly lower mean TST than non-asthma individuals. Among asthma cases, a quarter suffered from asthma-related sleep disturbances, and their mean TST was significantly lower than that of individuals without asthma. A comparison of mean TST between asthma cases suffering from sleep disturbances and those without revealed no significant difference; evaluation of the asthma phenotypes resulting in sleep disturbances also showed no significant difference in TST. Nonetheless, we found that there was a consistently lower mean TST among asthma cases suffering from sleep disturbances due to wheezing, dry nocturnal coughing, and nighttime asthma attacks. Additionally, a longer TST was significantly associated with a decreased likelihood of asthma, but this association was not observed when adjusted for asthma-related sleep disturbances. Indeed, shorter sleep duration and poorer SQ among asthmatics have been reported, and having a sleep duration of fewer than 5 h was significantly associated with increased asthma risk [78, 79]. Currently, we propose that shorter TST is likely mediated by asthma-related sleep disturbances, as opposed to TST being a risk factor for asthma.

The effects of various factors—physiological changes and physical posture associated with sleep, circadian clock regulation of immune function, and environmental conditions—have yet to be comprehensively teased apart [80]. Among the few studies assessing the influence of sleep on asthma, a prospective study found that adults with poor sleep habits had a higher risk of asthma within a follow-up period of at least 10 years, while experimental manipulation of sleep duration among asthma patients resulted in a decreased in peak expiratory flow rates and increased interference of activities by asthma symptoms [38, 81]. Nonetheless, while the directionality of the relationship between sleep and asthma remains unclear, the association of poor sleep with asthma has been well documented, with short sleep and poor sleep quality being associated with nocturnal respiratory symptoms [82, 83]. As with AD and AR, the circadian rhythm has been implicated: a circadian pattern of variation in airway inflammation has been observed [84]. In fact, variable airflow and bronchial hyperresponsiveness are characteristic of asthma, and asthma exacerbations frequently occur during the night [80, 85]. The coincidence of the circadian rhythms of several functions, resulting in the periodic increase in vagal tone, decreased epinephrine levels, and change in IgE/mast cell responses might play an important role in the manifestation and exacerbation of asthma [86, 87].

## Conclusion

We have established a baseline for the characteristics of sleep and allergic disease among young Chinese adults in Singapore. We note, however, the limitations of gauging sleep impairment using TST which is merely participants’ estimate of their nightly time spent asleep. A metric as precise as TST gives little detail regarding more descriptive sleep parameters, such as sleep-onset latency (SOL), wake time after sleep onset (WASO), or sleep fragmentation [88]. Moreover, our current analysis was unable to account for the possible effects of medication taken to treat allergic diseases—medications alleviating the effects of allergic diseases could have resulted in sleep less impacted by allergic disease symptoms, while sedative medications could have resulted in longer total sleep time or better sleep quality [89–92]. As such, our findings on the effect of sleep on allergic diseases or vice versa could have been underestimates. Thus, follow-up studies would be well-advised to consider the effect of medications on the interplay between sleep and allergic disease by collecting the necessary information from participants and adjusting for medication use in their analyses.

Although the sample size for each allergic disease was relatively small, we were able to identify associations between sleep and allergic diseases that were supported by the literature. Moreover, despite the cross-sectional nature of this study, we were able to obtain an indication of directionality by controlling for allergic disease-related sleep disturbances. Overall, while sleep quality was associated with AD, there was a lack of evidence to discern the direction of this relationship. Furthermore, we found that TST likely influences AR, and the impact of AR on sleep is primarily on SQ as opposed to TST. Finally, we provided evidence that the association between TST and asthma is likely mediated by asthma-related sleep disturbances. The present report thus constitutes the foundation for further studies investigating the relationship between sleep and allergic diseases. Future studies would be well-advised to use objective measurements of sleep, such as actigraphy or polysomnography methods which provide the capability of capturing the complexities of sleep. Furthermore, differential expression analysis of transcriptome samples obtained 12 h apart, in the day and at night, could be considered to identify differentially expressed genes which are linked to the circadian clock, immune function, or allergic disease. Functional characterization of any gene of interest could then be carried out to determine the mechanism by which the circadian clock regulates immune response.

### Supplementary Information

**Additional file 1: Supplementary Table 1.** Results from the logistic regression analyses for allergic disease manifestation against total sleep time only (model 1), sleep quality only (model 2), TST and sleep quality (model 3), and TST and sleep quality as interaction terms (model 4). **Supplementary Table 2.** Results from the logistic regression analyses for allergic disease manifestation against total sleep time only (model 1), sleep quality only (model 2), TST and sleep quality (model 3), and TST and sleep quality as interaction terms (model 4). In all models, allergic disease cases experiencing sleep disturbance due to allergic disease were excluded. **Supplementary Table 3.** Results from the logistic regression analyses for total sleep time against allergic disease only (model 1) and allergic disease and sleep disturbances due to allergic disease (model 2). **Supplementary Table 4.** Results from the logistic regression analyses for sleep quality against allergic disease only (model 1) and allergic disease and sleep disturbances due to allergic disease (model 2).

## Data Availability

All data used and included in this study are available from the corresponding author (F.T.C.).

## Abbreviations

95% CI: 95% Confidence interval
AD: Atopic dermatitis
AR: Allergic rhinitis
ARIA: Allergic Rhinitis and its Impact on Asthma
IgE: Immunoglobulin E
ipRGC: Intrinsically photosensitive retinal ganglion cells
ISAAC: International Study of Asthma and Allergies in Childhood
NUS: National University of Singapore
OR: Odds ratio
PSG: Polysomnography
RZR/ROR: Retinoid-related orphan nuclear hormone receptor family
SCN: Suprachiasmatic nuclei
SCORAD: SCORing Atopic Dermatitis
SMCGES: Singapore/Malaysia Cross-Sectional Genetic Epidemiology Study
SOL: Sleep onset latency
SQ: Sleep quality
TST: Total sleep time
WASO: Wakefulness after sleep onset
