# Supplementary material for: Sleep and allergic diseases among young Chinese adults from the Singapore/Malaysia Cross-Sectional Genetic Epidemiology Study (SMCGES) cohort
Source: J Physiol Anthropol. 2024 Jan 30;43:6. doi: 10.1186/s40101-024-00356-5 (PMC10826209; doi:10.1186/s40101-024-00356-5)
Supplement: Supplementary file 1 — Additional file 1: Supplementary Table 1. Results from the logistic regression analyses for allergic disease manifestation against total sleep time only (model 1), sleep quality only (model 2), TST and sleep quality (model 3), and TST and sleep quality as interaction terms (model 4). Supplementary Table 2. Results from the logistic regression analyses for allergic disease manifestation against total sleep time only (model 1), sleep quality only (model 2), TST and sleep quality (model 3), and TST and sleep quality as interaction terms (model 4). In all models, allergic disease cases experiencing sleep disturbance due to allergic disease were excluded. Supplementary Table 3. Results from the logistic regression analyses for total sleep time against allergic disease only (model 1) and allergic disease and sleep disturbances due to allergic disease (model 2). Supplementary Table 4. Results from the logistic regression analyses for sleep quality against allergic disease only (model 1) and allergic disease and sleep disturbances due to allergic disease (model 2). [file 40101_2024_356_MOESM1_ESM.docx]

**Supplementary table 1**: Results from the logistic regression analyses for allergic disease manifestation against total sleep time only (model 1), sleep quality only (model 2), TST and sleep quality (model 3), and TST and sleep quality as interaction terms (model 4).

| **Linear regression for increased total sleep time against the stated variables** |  | **Model 1:** TST only | | |  | **Model 2:** Sleep quality only | | |  | **Model 3:** TST and sleep quality | | |  | **Model 4:** interaction between TST and sleep quality | | |
| --- | --- | --- | --- | --- | --- | --- | --- | --- | --- | --- | --- | --- | --- | --- | --- | --- |
| **Allergic disease**  Variables |  | **OR** | **95% CI** | **p-value** |  | **OR** | **95% CI** | **p-value** |  | **OR** | **95% CI** | **p-value** |  | **OR** | **95% CI** | **p-value** |
| **Atopic dermatitis** |  |  |  |  |  |  |  |  |  |  |  |  |  |  |  |  |
| pTST (increase) |  | 0.961 | 0.862, 1.072 | 0.474 |  | NA | NA | NA |  | 0.951 | 0.823, 1.098 | 0.490 |  | 1.057 | 0.853, 1.309 | 0.613 |
| Sleep quality (Good - Very good) |  | NA | NA | NA |  | 0.817 | 0.612, 1.091 | 0.171 |  | 0.827 | 0.618, 1.105 | 0.199 |  | 3.210 | 0.425, 24.217 | 0.258 |
| pTST (increase) × Sleep quality (Good - Very good) |  | NA | NA | NA |  | NA | NA | NA |  | NA | NA | NA |  | 0.820 | 0.612, 1.099 | 0.184 |
| **Allergic rhinitis** |  |  |  |  |  |  |  |  |  |  |  |  |  |  |  |  |
| pTST (increase) |  | 0.905 | 0.82, 0.999 | 0.048 |  | NA | NA | NA |  | 0.937 | 0.821, 1.068 | 0.328 |  | 1.059 | 0.867, 1.293 | 0.574 |
| Sleep quality (Good - Very good) |  | NA | NA | NA |  | 1.011 | 0.776, 1.318 | 0.933 |  | 1.027 | 0.787, 1.34 | 0.847 |  | 4.648 | 0.731, 29.541 | 0.103 |
| pTST (increase) × Sleep quality (Good - Very good) |  | NA | NA | NA |  | NA | NA | NA |  | NA | NA | NA |  | 0.802 | 0.614, 1.048 | 0.106 |
| **Asthma** |  |  |  |  |  |  |  |  |  |  |  |  |  |  |  |  |
| pTST (increase) |  | 0.852 | 0.746, 0.972 | 0.017 |  | NA | NA | NA |  | 0.933 | 0.775, 1.123 | 0.462 |  | 1.062 | 0.808, 1.397 | 0.665 |
| Sleep quality (Good - Very good) |  | NA | NA | NA |  | 0.828 | 0.569, 1.205 | 0.325 |  | 0.842 | 0.577, 1.228 | 0.372 |  | 4.672 | 0.342, 63.858 | 0.248 |
| pTST (increase) × Sleep quality (Good - Very good) |  | NA | NA | NA |  | NA | NA | NA |  | NA | NA | NA |  | 0.778 | 0.532, 1.137 | 0.194 |

**Supplementary table 2**: Results from the logistic regression analyses for allergic disease manifestation against total sleep time only (model 1), sleep quality only (model 2), TST and sleep quality (model 3), and TST and sleep quality as interaction terms (model 4). In all models, allergic disease cases experiencing sleep disturbance due to allergic disease were excluded.

| **Linear regression for increased total sleep time against the stated variables** |  | **Model 1:** TST only | | |  | **Model 2:** Sleep quality only | | |  | **Model 3:** TST and sleep quality | | |  | **Model 4:** interaction between TST and sleep quality | | |
| --- | --- | --- | --- | --- | --- | --- | --- | --- | --- | --- | --- | --- | --- | --- | --- | --- |
| **Allergic disease**  Variables |  | **OR** | **95% CI** | **p-value** |  | **OR** | **95% CI** | **p-value** |  | **OR** | **95% CI** | **p-value** |  | **OR** | **95% CI** | **p-value** |
| **Atopic dermatitis** |  |  |  |  |  |  |  |  |  |  |  |  |  |  |  |  |
| pTST (increase) |  | 0.977 | 0.831, 1.148 | 0.779 |  | NA | NA | NA |  | 1.040 | 0.838, 1.291 | 0.720 |  | 1.066 | 0.766, 1.483 | 0.706 |
| Sleep quality (Good - Very good) |  | NA | NA | NA |  | 0.927 | 0.602, 1.429 | 0.732 |  | 0.916 | 0.592, 1.419 | 0.695 |  | 1.225 | 0.059, 25.556 | 0.896 |
| pTST (increase) × Sleep quality (Good - Very good) |  | NA | NA | NA |  | NA | NA | NA |  | NA | NA | NA |  | 0.959 | 0.619, 1.485 | 0.850 |
| **Allergic rhinitis** |  |  |  |  |  |  |  |  |  |  |  |  |  |  |  |  |
| pTST (increase) |  | 0.864 | 0.767, 0.973 | 0.016 |  | NA | NA | NA |  | 0.844 | 0.719, 0.991 | 0.038 |  | 0.966 | 0.754, 1.237 | 0.783 |
| Sleep quality (Good - Very good) |  | NA | NA | NA |  | 1.223 | 0.887, 1.687 | 0.22 |  | 1.279 | 0.923, 1.77 | 0.139 |  | 6.332 | 0.669, 59.936 | 0.108 |
| pTST (increase) × Sleep quality (Good - Very good) |  | NA | NA | NA |  | NA | NA | NA |  | NA | NA | NA |  | 0.789 | 0.569, 1.096 | 0.158 |
| **Asthma** |  |  |  |  |  |  |  |  |  |  |  |  |  |  |  |  |
| pTST (increase) |  | 0.879 | 0.754, 1.025 | 0.101 |  | NA | NA | NA |  | 0.967 | 0.781, 1.197 | 0.756 |  | 1.180 | 0.851, 1.637 | 0.322 |
| Sleep quality (Good - Very good) |  | NA | NA | NA |  | 0.86 | 0.562, 1.315 | 0.486 |  | 0.867 | 0.565, 1.331 | 0.515 |  | 10.803 | 0.504, 231.505 | 0.128 |
| pTST (increase) × Sleep quality (Good - Very good) |  | NA | NA | NA |  | NA | NA | NA |  | NA | NA | NA |  | 0.692 | 0.445, 1.077 | 0.103 |

**Supplementary table 3**: Results from the linear regression analyses for total sleep time against allergic disease only (model 1) and allergic disease and sleep disturbances due to allergic disease (model 2).

| **Linear regression for increased total sleep time against the stated variables** |  | **Model 1:** allergic disease only | | |  | **Model 2:** allergic disease and sleep disturbance due to allergic disease | | |
| --- | --- | --- | --- | --- | --- | --- | --- | --- |
| **Variables** |  | **Estimate** | **Standard error** | **p-value** |  | **Estimate** | **Standard error** | **p-value** |
| Atopic dermatitis |  | -0.044 | 0.061 | 0.474 |  | -0.036 | 0.089 | 0.687 |
| Sleep disturbances due to AD |  | NA | NA | NA |  | -0.014 | 0.108 | 0.901 |
| Allergic rhinitis |  | -0.11 | 0.055 | 0.047 |  | -0.157 | 0.065 | 0.016 |
| Sleep disturbances due to AR |  | NA | NA | NA |  | 0.127 | 0.091 | 0.164 |
| Ever asthma |  | -0.18 | 0.076 | 0.017 |  | -0.127 | 0.086 | 0.138 |
| Sleep disturbances due to asthma |  | NA | NA | NA |  | -0.212 | 0.161 | 0.188 |

**Supplementary table 4**: Results from the logistic regression analyses for sleep quality against allergic disease only (model 1) and allergic disease and sleep disturbances due to allergic disease (model 2).

| **Logistic regression for poor sleep quality against the stated variables** |  | **Model 1:** allergic disease only | | |  | **Model 2:** allergic disease and sleep disturbance due to allergic disease | | |
| --- | --- | --- | --- | --- | --- | --- | --- | --- |
| **Variables** |  | **OR** | **95% CI** | **p-value** |  | **OR** | **95% CI** | **p-value** |
| Atopic dermatitis |  | 1.224 | 0.917, 1.633 | 0.171 |  | 1.024 | 0.668, 1.572 | 0.912 |
| Sleep disturbances due to AD |  | NA | NA | NA |  | 1.339 | 0.799, 2.241 | 0.268 |
| Allergic rhinitis |  | 0.989 | 0.759, 1.288 | 0.933 |  | 0.771 | 0.562, 1.058 | 0.107 |
| Sleep disturbances due to AR |  | NA | NA | NA |  | 1.962 | 1.245, 3.089 | 0.004 |
| Ever asthma |  | 1.207 | 0.83, 1.757 | 0.325 |  | 1.118 | 0.733, 1.705 | 0.606 |
| Sleep disturbances due to asthma |  | NA | NA | NA |  | 1.395 | 0.606, 3.213 | 0.434 |
